# Development and validation of a protocol to determine product perception in relation to the moment of the day

**DOI:** 10.1016/j.mex.2025.103174

**Published:** 2025-01-16

**Authors:** M. Visalli, S. Plano, C. Tortorello, D. Vigo, M.V. Galmarini

**Affiliations:** a Centre des Sciences Du Goût et de l'Alimentation, CNRS, INRAE, Institut Agro, Université de Bourgogne, 17 rue Sully, Dijon 21000, France; b INRAE, PROBE Research Infrastructure, ChemoSens Facility, Dijon 21000, France; c Laboratorio de Cronofisiología, Universidad Católica Argentina, Argentina; d Member of CONICET (Consejo Nacional de Investigaciones Científicas y Tecnológicas), Argentina; e Facultad de Ingeniería y Ciencias Agrarias, Pontificia Universidad Católica Argentina (UCA), Argentina

**Keywords:** Chronobiology, Chronotype, Taste perception, Sleep, Moment specific Rate-All-That-Apply

## Abstract

Chronotype refers to an individual's tendency to engage in activities either earlier or later, in alignment with the biological rhythm of their body and its interaction with the environmental cycle. Chronotypes influence food preferences and meal timing, yet most studies rely solely on questionnaires without integrating real-time tasting data. To address this gap, we developed and validated a method to measure sensory perception and examine its variations throughout the day in alignment with circadian rhythms. Fifty-two university students completed the Munich Chronotype Questionnaire and, over four days within a week, they participated in sensory evaluations using a web-based questionnaire. At four daily time slots (morning, midday, afternoon, evening), participants tasted candies and assessed some sensory attributes—sweetness, sourness, bitterness, freshness, and overall flavor—using the Rate-All-That-Apply method. Before each evaluation, they also reported their level of hunger, thirst, tiredness, and willingness to complete the task. Reminders were sent via pre-programmed messages to ensure adherence to the schedule. The results demonstrate the feasibility of the method, with low attrition rates and consistent participant motivation over the study period. Sensory perception was found to vary across the day and in relation to chronotype, highlighting the method's potential for advancing research in sensory chrononutrition.

• A web-based questionnaire including tasting was developed to assess sensory perception at different times of the day over four days.
• Perception was analyzed in relation to chronotype.
• Face validity was confirmed, as significant variations based on chronotypes were observed.

## Graphical abstract

**Figure:**
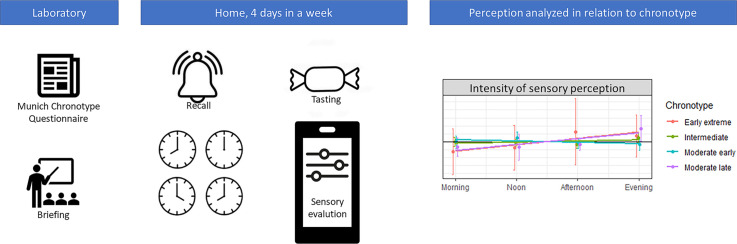

Specifications table

**Table:** 

Subject area:	**Food Science**
More specific subject area:	Sensory perception
Name of your method:	Moment specific Rate-All-That-Apply
Name and reference of original method:	Rate-All-That-Apply
Resource availability:	Dataset: https://doi.org/10.57745/4TFQTP

## Background

The biological clock regulates numerous physiological and behavioral variables: sleep-wake cycle, temperature, metabolism, hormonal secretions, etc. It prepares the organism to concentrate physical and cognitive capacities and eat during the day, and to rest and perform repair functions at night. Moreover, the control exerted by the biological clock over the daily ingestion-fasting cycle maintains a correct metabolic balance.

Chrononutrition, defined as the time of day in which foods are consumed in relation to the daily sleep-wake cycle, or activity-rest, is one of the main factors that determines health in an organism. It has been found that people who consume their main meal during the first part of the day have a lower body mass index (BMI) than those who consume them at night [[1], [2], [3]]. Likewise, different studies have shown that imbalances in the rhythms of ingestion-fasting and activity-rest contribute to obesity and metabolic syndrome, with shift work being one of the main causes of this ``metabolic jet lag'' [[4], [5], [6]]. While there is a trend towards the consumption of hypercaloric foods (rich in fats, carbohydrates, and sugars) at night [7], there are few studies that have empirically studied whether the time of day in which different energy sources are consumed is also due to changes in the way these are perceived. Some research has shown a relationship between lack of sleep and the perception of taste and smell [8,9]. Extreme conditions of sleep deprivation can induce cravings for unhealthy foods, while foods rich in umami or sour flavor can be experienced differently due to alterations in taste function. A reduction in olfaction has also been found in these conditions of complete lack of rest [10,11].

One of the main factors that influence the functions of our organism is the chronotype. Chronotype represents a person's preferences to start their activity earlier or later, responding to the biological temporal organization of the organism and how it relates to the environmental cycle. Thus, an individual with an extreme morning chronotype may have a difference of up to 2–3 h between their endogenous rhythms and environmental rhythms, compared to someone with an extreme evening chronotype [12]. In relation to food, it has been shown that there are differences in both the time of consumption of certain foods and the preference for certain foods between morning and evening chronotypes [13].

These results underscore the value of exploring the link between sensory perception and chronotypes, as previous studies relied on questionnaires rather than actual product tastings at various times of the day. To date, no standardized protocols or methods have been specifically designed to assess the perception of the same food at different times of the day in relation to individuals' circadian rhythms. Our objective was to fill this gap by developing a reusable method that can advance research in sensory chrononutrition.

## Method details

### Participants

To evaluate the potential of our method in detecting changes in perception, we focused on young students, a demographic often characterized by high stress levels and long hours spent indoors. Our sample was drawn from undergraduate students in their first, second, or third year of the Food Engineering, Environmental Engineering, Agronomic Engineering, and Industrial Engineering programs at the Faculty of Engineering and Agricultural Sciences, Pontificia Universidad Católica Argentina. The eligible population included 450 students. Based on an alpha error of 5 %, a beta error of 20 %, standard errors reported in the literature, and varying effect sizes [14], we estimated a required sample size of approximately 50 students. Recruitment was conducted through the university's online platforms and the faculty's social media channels. Additionally, physical notices were posted on bulletin boards in strategic campus locations, such as hallways, libraries, and cafeterias, to inform students about the study and how to participate. Exclusion criteria included students with a diagnosed sleep disorder (whether treated or untreated) and those with an aversion to or medical restrictions against consuming caramel, lemon, anise, or honey-mint candies.

A total of 52 students were included in the study. Data on demographics, including sex, age, body mass index, medical conditions, and associated risk factors, were collected. Additionally, general sleep health-related habits, such as time dedicated to physical activity, screen time, study hours, and sleep hours, were recorded. Specific information regarding sleep duration, phase, and quality was gathered using the Munich Chronotype Questionnaire (MCTQ, [15]).

### Samples evaluated by the participants

All samples were commercially available products. Hard candies were chosen for their practicality, their distinct flavors, the prominence of a specific sensory attribute in each, and their neutral association with any particular moment of the day. The selected candies and their salient sensory attributes were:

• Lemon-Sour (produced by Arcor, Argentina): sourness.
• Honey-Mint (produced by Arcor, Argentina): freshness.
• Caramel (produced by Arcor, Argentina): sweetness.
• Anise (produced by Mondelez, Argentina): bitterness.

### Data collection

Before starting the evaluations, participants were required to read and sign an informed consent form that had been previously approved by the ethics committee of the Pontificia Universidad Católica Argentina. To better understand taste perception, participants were characterized based on their sensitivity to bitterness by detecting PROP at three concentrations, following an adaptation of the method by Tepper et al. [16]. This approach focused exclusively on the perception of bitter taste. The prepared PROP solutions included the following concentrations: 0.032 mM (1.36 mg/L), 0.32 mM (13.6 mg/L), 3.2 mM (136 mg/L). This evaluation was conducted in a sensory laboratory (Laboratorio de Análisis Sensorial of the Pontificia Universidad Católica Argentina), in individual sensory booths designed to meet standard sensory evaluation guidelines.

After completing the bitterness sensitivity test, participants received four plastic bags, each containing four commercial candies in their original packaging. These candies were to be evaluated at home throughout the day, at different times, preferably after meals, during four specific time slots:

• Morning: 8:00–10:00 am
• Midday: 12:00–2:00 pm
• Afternoon: 4:00–6:00 pm
• Evening: 8:00–10:00 pm

Each participant completed a total of 16 evaluations, spread over four days in a same week, with one evaluation at each of the four different times of the day. Each participant consumed one candy of each type per day. The order of presentation was organized using a Latin square design to minimize order effects for each individual, while the sequence of candy types was randomized across participants to ensure balanced and unbiased evaluation. For each candy and at each time of the day, participants evaluated the intensity of the following attributes using a 5-point scale (0 = “not applicable”, 1 = “applies slightly”, 5 = “applies strongly”) following a Rate-All-That-Apply (RATA) approach [17]: sweetness, sourness, bitterness, freshness, total flavor. These attributes were chosen to represent three basic tastes, a trigeminal sensation, and a general flavor. All data were collected using the TimeSens software. Each participant accessed the platform through a unique, non-transferable link, secured with an individualized code and password, ensuring data integrity and confidentiality.

To ensure participants did not miss their scheduled evaluations, reminders were sent via pre-programmed WhatsApp messages, such as: *“Do not forget to evaluate the candy scheduled for this moment of the day. Here is your personal link.”* Participants could access the link on either their computer or smartphone, ensuring convenience and accessibility, as the specific time of day was critical for the study's objectives. If participants missed an evaluation during the designated time window, they were instructed to wait until the same time window the following day to resume the assessment. For example, if they missed the morning evaluation, they could only complete it the next morning. This strict adherence to the sample-moment of the day combination was emphasized repeatedly throughout the experiment to maintain the integrity of the study design.

Before each sensory evaluation, participants had to report their current state of hunger, thirst, tiredness, and willingness to complete the task. These states were recorded using 5-point scales. Hunger was rated from 1 (“Satiated/Full”) to 5 (“Hungry”). Thirst was rated from 1 (“Not thirsty at all”) to 5 (“Very thirsty”). Tiredness was rated from 1 (“Not tired/Active/Alert”) to 5 (“Very tired/Want to sleep”). Willingness to perform the activity was rated from 1 (“Not willing at all”) to 3 (“Indifferent”) and 5 (“Very willing”). In addition to evaluating the morning candy, participants were asked to complete a sleep journal. In this journal, they recorded the time they went to sleep the previous night, the time they woke up that day, and whether they took a nap the day before (if yes, they indicated the duration). This information had to be provided for all four days of the study.

Upon completion of the study, participants were given a small gift as a token of appreciation for their participation.

## Method validation

### Feasibility of the task

Among the 52 participants initially included, 46 completed the study, representing 88 % of the sample. Of these 46 participants, five had missing data: three participants missed data for one candy, and two participants had missing data for one evaluation of a candy.

**Fig. 1:**
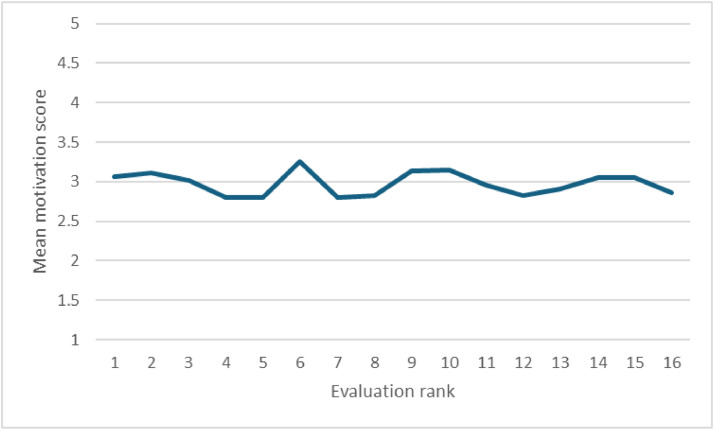
Changes in motivation throughout the experiment.

Fig. 1 shows that participants remained indifferent to the task throughout the 16 evaluations over the four consecutive days. While this result may be influenced by social desirability, it suggests that motivation did not decrease and that the experiment was not overly demanding.

### Preliminary data on chronotype-related differences

The MCTQ was employed to evaluate actual sleep times separately for work and free days. The *Mid-Sleep on Free Days corrected for sleep debt* (MSFsc, [18]) was calculated and used to categorize individuals according to their chronotype, which reflects their natural preferences for activity and rest periods. These categories are based on the corrected mid-sleep point, with scores typically grouped into distinct chronotype categories. *Early extreme* chronotypes, with an MSFsc before 3:00 AM, wake up very early and go to bed early. They are often most alert in the morning, with their energy levels declining quickly later in the day. *Moderate early* chronotypes, with an MSFsc between 3:00 and 5:00 AM, also favor morning activity but have slightly less pronounced early habits. *Intermediate chronotypes*, with an MSFsc between 5:00 and 7:00 AM, constitute the majority of the population. They have no strong preference for morning or evening and tend to peak in energy during the middle of the day. *Moderate late* chronotypes, with an MSFsc between 7:00 and 9:00 AM, are more active and alert in the evening, preferring later bedtimes and wake-up times.

To validate whether our method effectively captured variations in sensory perception across different times of the day, in relation to chronotype, we performed several analyses. To evaluate intra-subject variability across different times of day (morning, noon, afternoon, evening), intensity scores for each attribute were centered by subject and product. The standard deviations of these intensity scores were then calculated by subject, product, and attribute, and correlated with individual MSFsc values to examine the influence of chronotype on intra-subject variability in sensory perception. A Pearson's correlation coefficient of −0.15 (*p* = 0.030) was observed, indicating a small but significant relationship between intra-subject variability and MSFsc.

To further investigate the impact of chronotype on sensory perception, the mean and confidence intervals of centered intensity scores were plotted across different periods, along with trend lines over these periods.

**Fig. 2:**
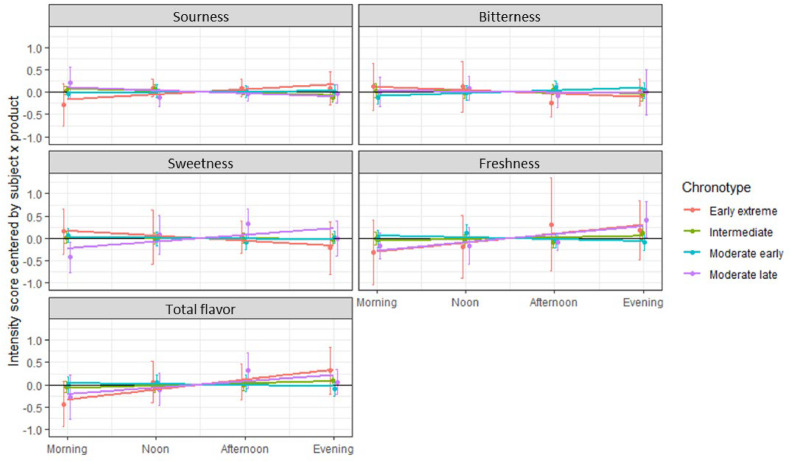
Variations in perception by moment of day and chronotype.

Fig. 2 indicates that the intensity of sensory perception for each attribute was influenced by chronotype, with more pronounced variations observed in *early extreme* and *moderate late* chronotypes, especially during the morning and evening periods.

Table 1 presents the estimates from the linear regression models, highlighting several variations potentially dependent on the time of day. For example, the intensity of *Total flavor* significantly increased over periods (α = 0.10) for all chronotypes except for *moderate early*. Some variations appeared specific to certain chronotypes, such as the intensity of *Sweetness* and *Freshness*, which significantly increased over periods exclusively for *moderate late* chronotypes. The variation in *Sourness* intensity showed contrasting trends depending on the chronotype, increasing over periods for *early extreme* chronotypes but decreasing for *intermediate* ones - the latter being the only intensity to show a significant decline over time.

**Table 1:** Estimates (p-values) of the linear regression model centered intensity score = moment + error.

Chronotype	Sourness	Bitterness	Sweetness	Freshness	Total flavor
Early extreme	↗ 0.11 (0.084)	→ −0.07 (0.374)	→−0.11 (0.254)	→ 0.20 (0.184)	↗ 0.22 (0.017)
Moderate early	→ 0.01 (0.665)	↗ 0.06 (0.087)	→ −0.02 (0.559)	→ −0.04 (0.320)	→ −0.02 (0.602)
Intermediate	↘ −0.05 (0.034)	→ −0.02 (0.462)	→ −0.01 (0.616)	→ 0.04 (0.248)	↗ 0.05 (0.053)
Moderate late	→ −0.07 (0.184)	→ −0.02 (0.816)	↗ 0.15 (0.067)	↗ 0.18 (0.012)	↗ 0.14 (0.082)

The differences in sensory intensity trends across chronotypes align with known circadian principles, where physiological and sensory functions vary with time. The pronounced variations for *early extreme* and *moderate late* chronotypes in the morning and evening are consistent with their respective peak activity periods. Variations in *Sourness* intensity based on chronotype suggest potential interactions between individual circadian rhythms and the perception of specific sensory attributes, which merit further investigation. The increasing trend in *Total flavor* across periods for most chronotypes might indicate a general temporal pattern in sensory sensitivity or accumulation effects. Similarly, the specific changes in *Sweetness* and *Freshness* for *moderate late* chronotypes could reflect unique sensory processing tied to their evening-oriented rhythm.

Our experimental design and measurement approach successfully captured variations in sensory perception across different times of the day. The observed temporal trends in sensory intensity, such as the increase in *Total flavor* for most chronotypes and the contrasting patterns in *Sourness* perception depending on chronotype, demonstrate the sensitivity of our methodology to detect both general and specific changes in perception. This indicates that the combination of repeated measurements, the alignment of sensory evaluations with specific time periods, and the consideration of chronotype as a variable allowed us to reveal meaningful fluctuations in sensory response throughout the day. These results validate our approach in capturing the dynamic nature of sensory perception.

## Limitations

This study was designed as a preliminary investigation to test the feasibility of capturing variations in sensory perception across different times of the day. While our preliminary results demonstrate that the experimental design and measurement methods are capable of detecting temporal and chronotype-specific trends, they should not be interpreted as definitive findings. The primary aim was to evaluate the methodological approach and its potential for future studies, rather than to establish firm conclusions about sensory perception variations. Notably, the small sample size and underrepresentation of extreme chronotypes could have influenced the results, and the observed variations might also reflect random fluctuations due to the non-repeatability of the evaluations or other confounding factors. Future research should involve larger, more balanced samples and incorporate measures to enhance repeatability and control for potential confounders.

## Ethics statements

The project was analysed and approved by the ethics committee in research (Comité de Ética en Investigación, CEI) of the Pontificia Universidad Católica Argentina. The research was carried out following the Declaration of Helsinki. The participants were informed of the conditions for participating and they gave informed consent validating a written form. They were able to withdraw from the study at any time without giving a reason. The products tested were commercial products, safe for consumption.

## CRediT authorship contribution statement

**M. Visalli:** Conceptualization, Software, Validation, Formal analysis, Data curation, Writing – review & editing, Visualization. **S. Plano:** Conceptualization, Methodology, Validation, Investigation, Resources, Data curation, Writing – review & editing, Visualization, Supervision, Funding acquisition. **C. Tortorello:** Conceptualization, Methodology, Investigation, Data curation, Writing – review & editing. **D. Vigo:** Conceptualization, Methodology, Visualization, Writing – review & editing, Funding acquisition. **M.V. Galmarini:** Conceptualization, Methodology, Validation, Investigation, Resources, Data curation, Writing – review & editing, Visualization, Supervision, Project administration, Funding acquisition.

## Declaration of competing interest

The authors declare that they have no known competing financial interests or personal relationships that could have appeared to influence the work reported in this paper.

## Data Availability

I have share the link to the dataset.
